# *In Vitro* Antibacterial, Antioxidant, Cytotoxicity Activity, and *In Silico* Molecular Modelling of Compounds Isolated from Roots of *Hydnora johannis*

**DOI:** 10.1155/2024/3713620

**Published:** 2024-06-21

**Authors:** Teshome Degfie, Milkyas Endale, Muhdin Aliye, Rajalakshmanan Eswaramoorthy, Tariku Nefo Duke, Aman Dekebo

**Affiliations:** ^1^Department of Applied Chemistry, Adama Science and Technology University, P.O. Box 1888, Adama, Ethiopia; ^2^Department of Chemistry, Dire Dawa University, P.O. Box 1362, Dire Dawa, Ethiopia; ^3^Traditional and Modern Medicine Research and Development Directorate, Armauer Hansen Research Institute, P.O. Box 1005, Addis Ababa, Ethiopia; ^4^Department of Biomaterials, Saveetha Dental College and Hospitals, Saveetha Institute of Medical and Technical Sciences, Saveetha University, Chennai 600 077, India; ^5^Department of Materials Science and Engineering, National Taiwan University of Science and Technology, Taipei, Taiwan; ^6^Institute of Pharmaceutical Sciences, Adama Science and Technology University, P.O. Box 1888, Adama, Ethiopia

## Abstract

The plant *Hydnora johannis* has been utilized in folk medicine. Analyzing phytochemical composition of dichloromethane/methanol (1 : 1) root part of *Hydnora johannis* gave oleic acid (**1**), caffeic acid-2-hydroxynonylester (**2**), catechin (**3**), and a pregnane derivative (**4**). NMR spectroscopy was used to characterize compounds **1–3**, while compound **4** was identified through GC-MS analysis and literature comparison. The cytotoxicity of extracts from roots of *H. johannis* was conducted against MCF-7 cell lines (human breast cancer) by MTT assay. According to the cytotoxicity study, *n*-hexane extract exhibited a high level of toxicity with 28.9 ± 5.6% cell viability. Antibacterial activity was tested against *Escherichia coli*, *Pseudomonas aeruginosa*, *Staphylococcus aureus*, and *Streptococcus pyogen.* The highest bacterial growth mean inhibition zone was measured for catechin (3) (13.72 ± 0.05 mm)) against *P. aeruginosa* at 0.25 mg/mL and acceptable related to standard. Antioxidant activity was studied by the DPPH assay. Based on the data from the antioxidant study, DCM/MeOH extract (70.32%) and catechin (**3**) showed good antioxidant activity (65.61%) (IC_50_ 0.25 *μ*g/mL) relative to that of the positive control (78.21%, IC_50_ 0.014 *μ*g/mL) at 12.5 *μ*g/mL. In each docking pose, catechin (**3**) scored higher binding affinity of −7.9, −7.2, and −6.4 kcal/mol towards PqsA, DNA gyraseB, and *S. aureus* PK, respectively, compared to amoxicillin (−8.1, −6.1, and −6.4 kcal/mol). All five Lipinski rules were obeyed by compounds **1–3**, which showed an acceptable drug resemblance. The lipophilicity was computed as less than five (1.47–4.01) indicating a lipophilic property. Catechin (**3**) obeys Veber's rule implying its good oral bioavailability. Binding affinity scores of catechin (**3**)-protein interactions are in line with those from *in vitro* tests, indicating its potential antibacterial effect. The obtained cytotoxicity and antibacterial activity results support the utilization of *H. johannis* in folk medicine.

## 1. Introduction

Genus *Hydnora* (Hydnoraceae) includes hypogenous parasitic plant species with a massive root system and reduced morphological features [1, 2]. It is widely distributed in the region of southern Africa, across East Africa to the Arabian Peninsula [3, 4]. Eight species of genus *Hydnora* [5] were described. The plant species of genus *Hydnora* has been used as medicine for both human and livestock [6, 7]. Bioactive phytochemical constituents including flavonoids, tannins, terpenoids, saponins, and alkaloids have been reported from genus *Hydnora* [2, 8]. *Hydnora johannis* Becc. (synonymous: *H. abyssinica* A.Br.) is the widely spread species in the African continent. It parasitizes on members of genus *Acacia*, *Albizia*, and *Kigelia Africana* plants [1, 9]. In Ethiopia, *H. johannis* occurs dominantly in Shewa, Sidamo, Bale, Arsi, and Hararge, and is known by its local name DechMerech (Afan oro/Amh), Lipti (Som) [10]. The root extracts are used to treat ailments including hemorrhoid, swollen abdomen, cancer, diabetes, dysentery, and kidney and bladder complaints [6]. In Sudan, the water extract is given for healing tonsillitis and dysentery [11]. Traditional medicine practitioners in Ethiopia recommend the plant as a therapeutic agent for swelled breast [10], painful swelling (gofla, Afan Oromo), hemorrhage [12], cancer, tumors, and inflammatory ailments [13]. Due to the fact that the plant has prevalent ethnomedicinal uses to treat various diseases, more studies need to be developed about the chemical constituent and biological activities of the plant *H. johannis*. In this sense, this study seeks to examine cytotoxicity, antibacterial, and antioxidant properties of extracts, and molecular modelling of compounds from CH_2_Cl_2_/CH_3_OH root extract of *H. johannis*.

## 2. Materials and Methods

### 2.1. Research Procedure

Maceration was used to obtain crude extracts. On precoated silica gel GF254 (Merck) aluminum sheet, analytical thin layer chromatography (TLC) was performed. A UV cabinet with a lamp of 254 and 365 nm was employed to visualize TLC spots, iodine vapor, and vanillin spray. A column was set on silica gel with 60–120 mesh size. The NMR data were obtained on Bruker Avance 400 MHz spectrometer. Both, GC/MS: coupled GC (7890B, USA) and MS (5977A Network), were obtained from Agilent Technologies. All materials used met the required standard.

### 2.2. Preparation of Plant Materials

Root part of *H. johannis* was obtained from Huruta, Arsi Zone, Oromia, Ethiopia, Jan 2020. After being confirmed by the chief technician, Botanist Melaku Masresha, kept at the National Herbarium, AAU, Ethiopia, with voucher code THJ10/17.

### 2.3. Isolation of Pure Compounds

Five hundred grams of air-dried and powdered roots of *H. johannis* has been consecutively extracted by *n*-hexane, CH_2_Cl_2_/CH_3_OH, and CH_3_OH for 72 h each, three times at normal temperature. Each filtrate was subjected to a rotary evaporator at 40°C to produce crude extracts weighing 22 g, 35 g, and 30 g, respectively. Ten grams of CH_2_Cl_2_/CH_3_OH (1 : 1) crude extract was loaded to fractionation with rising polarity of *n*-hexane/EtOAc and then by DCM/MeOH. Inspecting the TLC profile gave about 120 fractions, each 50 mL. Fractions 31–40 were combined and purified in a small column with an isocratic mode with 5% EtOAc/*n*-hexane and yielded compounds **1** (42 mg) and **2** (56 mg). Fractions 67–77 which fractionated with 20% *n*-hexane in EtOAc, further purified using a small column with 0.5–2% MeOH in DCM and afforded compound **3** (50 mg). Fractions 19–26 which were collected with 25–30% ethyl acetate in hexane yielded compound **4** (32 mg).

### 2.4. Cytotoxicity Test

The antiproliferative effect of *n*-C_6_H_14_, CH_2_Cl_2_/CH_3_OH, and CH_3_OH extracts from roots of *H. johannis* was conducted against MCF-7 cell lines (human breast cancer) [14], through MTT assay [15, 16]. The MCF-7 cells were acquired from NCCS, Pune, India, grown at 37°C in DMEM containing 10% FBS and 1% antibiotics (100 U·mL^−1^ penicillin and 100 *μ*g·mL^−1^ streptomycin) in a moisten air having 5% CO_2_. Confluent cells were separated using the trypsin-EDTA solution, and 5000 cells per well were used for subculture. At 50% confluence, the media was evacuated, and the cells exposed to 20 *μ*g of the plant extract dissolved in DMSO (20 *μ*L) and set at 37°C for 24 h in a CO_2_ incubator. Finally, samples were incubated with MTT (4 mg·mL^−1^) for 3 h. With the use of a typical microplate reader, absorbance was determined at 540 nm. Percentage cell viability was managed to express cytotoxicity. The findings are shown as the average of three replicated experiments. The equation for calculating cell viability is as follows:

(1) $$\begin{array}{c} \text{Percent cell viability}=\frac{\text{absorbance of treated}}{\text{absorbance of control}}\times 100\%. \end{array}$$

### 2.5. Antibacterial Activity Test

#### 2.5.1. Bacterial Strains

The *Escherichia coli* ATCC 25922, *Pseudomonas aeruginosa* ATCC 27853, *Staphylococcus aureus* ATCC 25923, and *Streptococcus pyogen* ATCC 19615 strains were made available by the Ethiopian Public Health Institute (EPHI). These strains are among the drug-resistant pathogenic strains of most of the first-line drugs [17], and any substance toxic to these strains is also accepted to be active on other strains [18]. Experiments were carried out at the microbiology laboratory of Adama Science and Technology University.

#### 2.5.2. Antibacterial Activity Test

The studied compounds (**1–3**) were tested for their bacterial growth inhibition. Disc-diffusion assay based on previously reported protocol was employed [19, 20]. Inoculums for antibacterial testing were prepared by transferring nearly 2–4 fresh strains with identical morphology into a saline solution. The turbidity of each bacterial suspension was corrected according to a 0.5 McFarland solution (10^8^ CFU/mL) [21]. Fresh bacterial suspension of each strain was streaked uniformly onto separate Petri dish holding Mueller–Hinton agar (HiMedia) medium swapping with sterile cotton swap. Stock solutions (5 mg in 5 mL) of samples were made in 4% DMSO, followed by preparation of 0.5 and 0.25 mg/mL of samples (**1–3**) from corresponding stocks. Sterilized paper discs with 6 mm were prepared and loaded with the sample solution of 100 *µ*L/disc, placed on the surface of the MHA plate, and incubated at 37°C for 18–24 hr. Standard antibiotic disc of amoxicillin was considered as positive and DMSO as negative controls. The area of microbial growth inhibition was computed by measuring the diameter (in mm) of the clear area surrounding the paper disc [20, 22]. Trails were carried out twice, and using SPSS (version 20) statistical software, the outcomes were analyzed and reported as mean ± SD.

### 2.6. Antioxidant Activity

The oxidant trapping property of studied samples was performed based on the previously reported DPPH assay [23]. Using serial dilution, samples of 200, 100, 50, and 25 g/mL were created, added to a newly made 0.04% DPPH solution in methanol, and incubated at 37°C, and absorbance at 517 nm was recorded. In conditions similar to those of test samples, we set ascorbic acid as a positive control. The radical trapping ability in percent for each substance was determined using the following formula [23]:

(2) $$\begin{array}{c} \text{Percentage inhibition}=\frac{\left(A_{\text{control}}-A_{\text{sample}}\right)}{A_{\text{control}}}\times 100\%, \end{array}$$

where *A*_control_ represents absorbance of the DPPH solution and *A*_sample_ represents absorbance of the test sample. In addition, radical trapping of studied samples was given as IC_50._

### 2.7. Molecular Modelling

We performed docking studies to understand the ligand-protein interaction at a molecular level and to reveal the cause of such interactions. Isolated compounds **2** and **3** were separately put into an active binding site of target proteins including PqsA (5OE5), *DNA* gyraseB (*6F86*), and *S. aureus* PK (3T07) [24, 25]. AutoDock Vina with previously reported protocol [24] applied to perform docking. ChemOffice program (Chem Draw 16.0) was employed to depict the structure of ligand molecules and assigned the correct 2D scheme. The reduced energy of ligands was monitored by Chemdraw3D. Conformations with reduced energy level were sent into AutoDock Vina to undergo binding simulations. Receptor proteins (6F86, 5OE5, and 3T07) crystal structures were retrieved from the PDB.

Using formerly reported standard technique [26], the proteins were designed by eliminating co-existing groups and certain H_2_O. The selected enzyme file was then produced by AutoDock version 4.2 (MGL tools 1.5.7) leaving related residue with protein. Initializing the molecule mainly includes the addition of hydrogen atoms and the addition of the Kolman charge to the protein molecule, while, for the ligand molecule, it also includes the addition of the Gasteiger charge, identifying aromatic carbons, and detecting rotatable bonds. In the docking process, a grid box was constructed using 20 × 20 × 20, pointing in *x*, y, and *z* directions, respectively, with a grid point spacing of 0.375 A°. The center grid box is of 29.09 × 22.86 × 30.06 A°, 61.68 × 28.33 × 64.29 A°, and −44.36 × −25.35 × −11.00 A° for 5OE5, 6F86, and 3T07, respectively. Docking genetic algorithms conditioned on AutoDock Vina and managed to find the best docked configuration of a ligand to a protein. Nine conformations for each ligand were taken into account, and PyMOL and Discovery Studio Visualizer [27] managed to select the one with the minimum binding energy to discuss the interaction.

### 2.8. Drug-Likeness and ADMET Properties

The concept of drug resemblance and ADMET properties were designed to disclose important guidelines for initial phases of drug research to enhance the likelihood that a compound will enter clinical trial stages [26]. To estimate the drug-likeness and pharmacokinetic parameters, initially, the structure of pure compounds was transformed into their canonical SMILES, then subjected to PreADMET and SwissADME [28]. Lipinski's rule [29] and Veber's filter [30] were used to estimate drug-likeness features.

Main ADME parameters [31], such as GIA, skin permeability (logKp) levels, brain access (BBB), permeability glycoprotein (P-gp) substrate/inhibitor, and cytochromes (CYP) inhibition, were computed. CYP P450 inhibition plays a major role in pharmacokinetics-related drug-drug interactions which can result in harmful or related effects attributed to reduced removal/accumulation of drugs or metabolites [28]. BOILED-Egg plot was set to evaluate the human intestinal absorption, brain access, and P-gp score for the isolated entity [32]. The toxicity profile including the level of toxicity (LD50, mg/kg)/toxicity class, and other toxicities were predicted using ProTox-II server [33].

## 3. Results and Discussion

**Figure 1:**
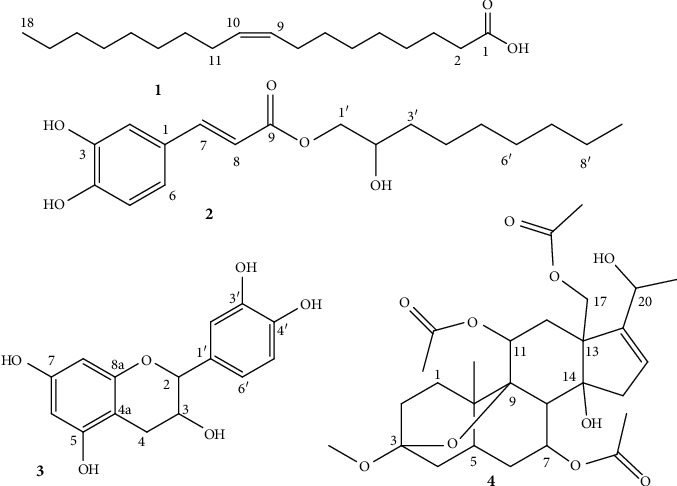
Compounds' structure obtained up on fractionation of the root of *H. johannis*.

Purification of CH_2_Cl_2_/CH_3_OH (1 : 1) extract of the root of the plant under investigation by chromatography on silica gel resulted from four compounds (Figure 1). NMR spectroscopy and GC/MS data were analyzed by comparing those with the literature data to determine the structure of the studied compounds.

Compound **1** was obtained as a solid (18 mg) with mp of 13.5–14°C and the *R*_*f*_ value was determined as 0.47 (9 : 1 *n*-hexane/EtOAc). Its proton spectrum showed a multiplet signal at *δ* 0.99 assigned to -CH_3_ protons (H-18) (Table 1, Figure S1). Whereas the signals at *δ* 5.33, *δ* 1.83 (m), and *δ* 2.31 (m) are assignable to olefinic methine protons (H-9, 10), allylic methylene protons (H-8, H-11), and methylene protons adjacent to carbonyl carbon, respectively. Together with the aforementioned data, the ^13^C (100 MHz, CDCl_3_, Figure S2) and DEPT-135-NMR (Figure S3), and comparing with the literature values [34], compound **1** was identified to be oleic acid (Figure 1).

Compound **2** was isolated as an amorphous material with *R*_*f*_ of 0.48 (eluent *n*-hexane/ethylacetate) (1 : 1). The proton spectrum (Figure S4) exhibited signals for aromatic H with the ABX system at *δ* 7.08 (d, *J* = 8.1 Hz), *δ* 7.05 (d, *J* = 2.0 Hz) and *δ* 6.92 (d, *J* = 8.1 Hz), and *trans*-olefinic protons at *δ* 7.62 (d, *J* = 15.9 Hz) and *δ* 6.30 (d, *J* = 15.9 Hz) (Table 2). The signals at *δ* 4.34 and 4.28 (each dd, *J* = 12.0, 4.4 Hz, 2H) and *δ* 4.15 (m, 1H) belong to sp^3^ oxy-methylene protons (H-1′) and oxy-methine (H-2′) groups. Whereas signals at *δ* 0.88 (t, *J* = 6.8 Hz, 3H) and *δ* 1.62–2.37, respectively, belong to terminal methyl (C-9′) and -CH_2_ (H-3′ to H-8′). The ^13^C NMR (100 MHz, CDCl_3_, Figure S5) and DEPT-135 (Figure S6) displayed aromatic quaternary signals at *δ* 147.9 (C-4), 146.7 (C-3), and 130.0 (C-1), aromatic -CH protons at *δ* 114.7 (C-2), 115.8 (C-5), and 123.0 (C-6), olefinic carbons at *δ* 144.7 (C-7) and 115.6 (C-8), carbonyl group at *δ* 167.5 (C-9) (Table 2). Similarly, oxygenated sp^3^ -CH_2_ and -CH were exhibited at *δ* 68.3 (C-1′) and 65.0 (C-2′). Methylene carbons appeared at *δ* 34.1, 31.9, 29.1, 27.1, 24.8, and 22.7 (C-3′ to C-8′), whereas terminal methyl was observed at *δ* 14.1 (C-9′). The obtained spectrum data and the data given in the literature [35] confirm that compound **2** is caffeic acid-2-hydroxynonyl ester, **2**, Figure 1).

Compound **3** exhibited the *R*_*f*_ value of 0.32 with mobile phase of CH_2_Cl_2_/CH_3_OH (9 : 1). Its proton spectrum (Figure S7) displayed aromatic proton signals at *δ* 6.74 (d, *J* = 1.9 Hz), 6.68 (d, *J* = 8.1 Hz), and 6.65 (dd, *J* = 8.2, 1.9 Hz) with the ABX spin pattern. In the same region of the spectrum, two aromatic protons with AB spin pattern were also observed at *δ* 5.84 (d, *J* = 1.2 Hz) and 5.81 (d, *J* = 1.2 Hz). On the other hand, two oxygenated sp^3^ methine signals were observed at *δ* 4.46 (d, *J* = 7.9 Hz), 3.88 (m) along with diastereotopic methylene signals connected to sp^2^ carbon at *δ* 2.81 (dd, *J* = 16.0, 5.5 Hz, H-4a), and 2.41 (dd, *J* = 16.0, 5.5 Hz, H-4b) (Table 3, Figure S7). The aforementioned proton spectrum and the ^13^C NMR (Figure S8) together with its DEPT-135 (Figure S9) suggest a flavan skeleton with AB pattern in ring A, ABX pattern in ring B, hydroxy group at C-3 of ring C which is in line with formerly described result of catechin (**3**, Figure 1). From the same plant species, Yagi et al. [6] reported catechin (**3**) with its antioxidant and antidiabetic activities [6]. This compound was previously reported from *Dioscorea bulbifera* L. Tubers with promising hypoglycemic activity [36, 37] and roots of *Embelia schimperi* were reported with promising antibacterial properties towards *S. aureus* and *E. coli* [38].

**Figure 2:**
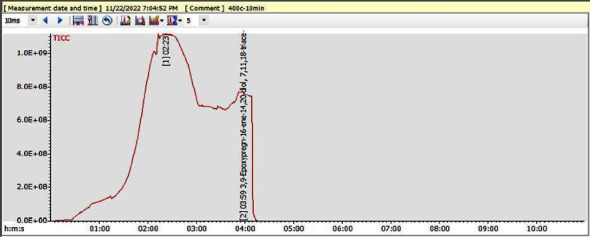
GC-MS chromatogram of compound **4**.

**Figure 3:**
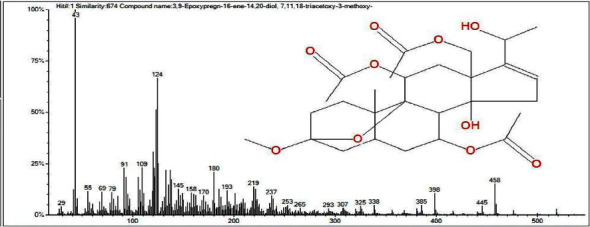
Mass spectrum and structure of compound **4.**

The GC-MS analysis of fractions 19–26 (25–30% EtOAc in *n*-hexane as eluent) showed the presence of two peaks as shown in the chromatogram (Figure 2). By comparing the mass spectra (Figure 3) of the composition at the retention time of 4 min with the NIST library, compound **4** was identified as a pregnane derivative named as 3,9-epoxypregn-16-ene-14,20-diol,7,11,18-triacetoxy-3-OCH_3_, previously reported from the GC-MS of CH_3_OH extract of *Cyclamen persicum* [39].

### 3.1. Cytotoxicity Tests

Cytotoxicity of *n*-C_6_H_14_, CH_2_Cl_2_/CH_3_OH, and CH_3_OH extracts of the root of *H. johannis* were studied against MCF-7 cell line at 20 *µ*g/mL samples' concentrations.  Table 4 displayed the percentage of cell viability. According to the result, *n*-hexane extract exhibited strong cytotoxicity against MCF-7 with % cell viability of 28.9% (Table 4). Whereas the methanol and DCM/MeOH (1 : 1) and MeOH extracts exhibited moderate cytotoxicity with percentage viability of 51.6% and 59.5%, respectively. Based on the obtained result, the *n*-hexane extract (20 *μ*g/mL) exhibited % cell viability <50% which is acceptable as stated by the NCI criteria (estimated IC_50_ less than 30 *μ*g/mL) [40]. The cytotoxicity result showed that the evaluated extracts exhibited a promising cytotoxicity effect against the MCF-7 cell line, which support the traditional importance of *H. johannis* for breast cancer treatment.

### 3.2. Antibacterial Activity

According to previous study result [41], the extract of dichloromethane/methanol was found to have better activity than *n*-hexane and methanol extracts towards *P. aeruginosa*, *E. coli*, and *S. aureu*s. In this study, the abilities of compounds **1**, **2**, and **3** to stop bacterial growth, each at 0.25 and 0.50 mg/mL, were evaluated and reported in the zone of inhibition (ZI). The mean zone inhibition (ZI in mm) results are displayed in Table 5.

According to the antibacterial study result, at 0.25 mg/mL, compound **3** scored the largest ability to stop the growth of all tested strains (Table 5), scoring growth preventing diameter of 13.72 ± 0.05, 12.25 ± 0.1, and 11.62 ± 0.12 mm towards *P. aeruginosa*, *E. coli*, and *S. pyogen*, respectively. Relative to the standard (16.0 ± 0.0 mm, 15.25 ± 0.25 mm, and 16.25 ± 0.25 mm, respectively), this result was found to be good. Caffeic acid-2-hydroxynonylester (**2**) displayed the IZ value of 10.32 ± 0.12 mm, 9.19 ± 0.06 mm, 8.97 ± 0.22 mm, and 8.2 ± 0.3 mm against *P. aeruginosa*, *E. coli*, *S. aureus*, and *S. pyogen*, respectively. Oleic acid (**1**) displayed the moderate zone of inhibition of 7.89 ± 0.05 mm against *P. aeruginosa*. The experimental result showed that catechin (**3**) displayed a considerable activity that goes in line with traditional uses of *H. johannis* to treat infectious diseases.

### 3.3. Antioxidant Activity

**Figure 4:**
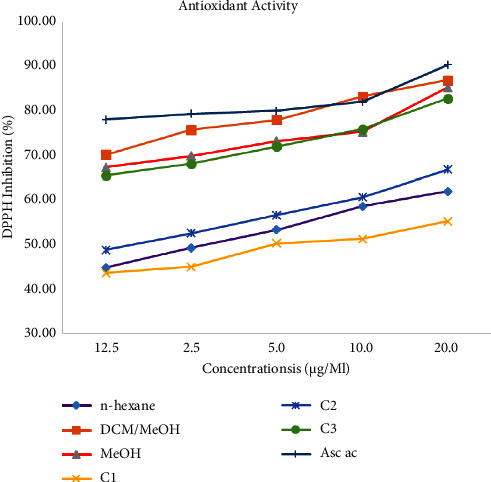
Percentage DPPH trapping ability of compounds **1**–**3** and the standard.

The free radical trapping ability of compounds **1**, **2,** and **3**, and extract was evaluated using the DPPH method [23]. As per the result, studied extracts and three compounds trap free radicals based on the administered amount (Table 6, Figure 4). At 12.5 *μ*g/mL, the DCM/MeOH extract exhibited the largest DPPH trapping ability (70.32%) with IC_50_ of 1.44 *μ*g/mL, being followed by the MeOH extract (67.44%). At the same lowest concentration, among the tested compounds (**1**–**3**), the largest radical trapping ability was shown by catechin (**3**) (65.61%) at IC_50_ of 0.25 *μ*g/mL, relative to the positive control (78.21%) at IC_50_ of 0.014 *μ*g/mL. Caffeic acid-2-hydroxynonylester (**2**) displayed considerable scavenging activity (48.9%), with an IC_50_ value of 1.31 *μ*g/mL, whereas oleic acid (**1**) displayed lower scavenging activity (43.64%) relative to standard (78.21%). The ability of catechin (**3**) to trap free radicals suggests the use of this compound as a natural antioxidant (Table 6, Figure 4), supported by the drug-likeness *in silico* computation study of the compound with higher NHD and NHA values.

### 3.4. Molecular Docking Study

**Figure 5:**
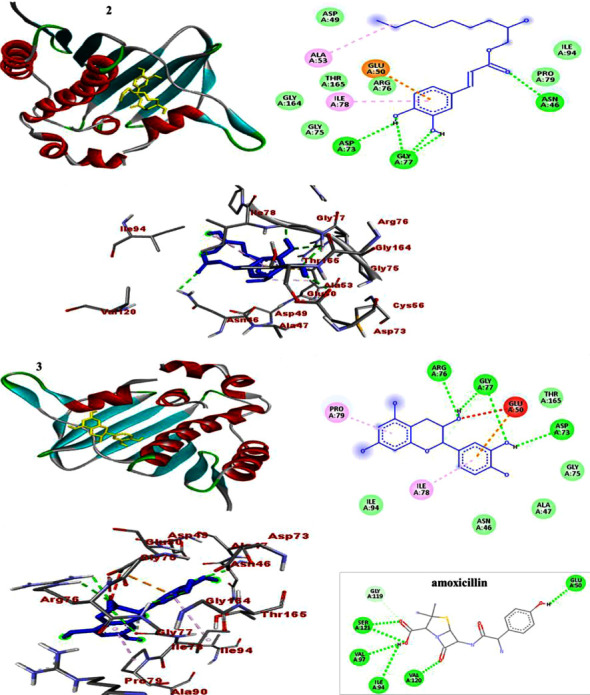
The 2D and 3D binding interactions of caffeic acid-2-hydroxynonyl ester (**2**), catechin (**3**), and amoxicillin against DNA gyraseB (PDB ID: 6F86).

**Figure 6:**
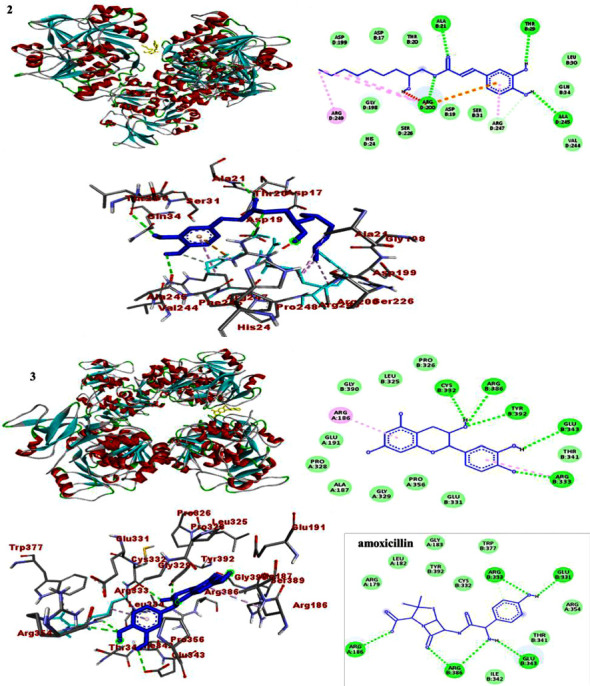
The 2D and 3D binding interactions of caffeic acid-2-hydroxynonyl ester (**2**), catechin (**3**), and amoxicillin against PqsA (PDB ID: 5OE3).

**Figure 7:**
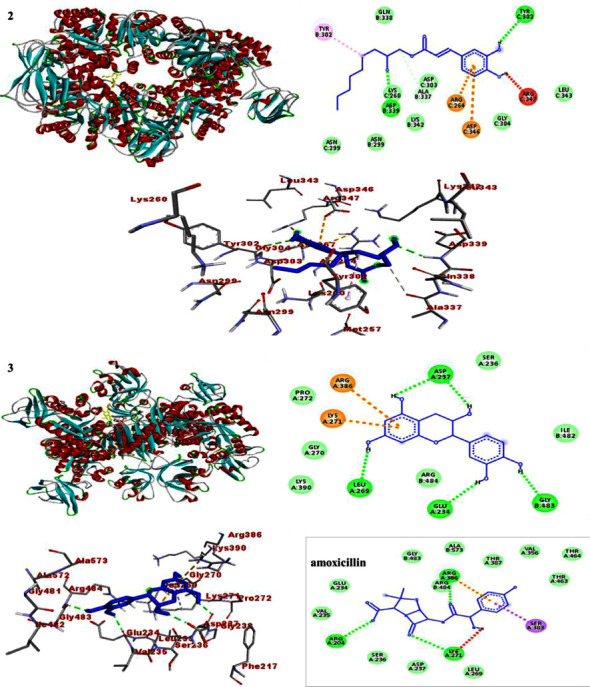
The 2D and 3D binding interactions of caffeic acid-2-hydroxynonyl ester (**2**), catechin (**3**), and standard against *S. aureus* PK (PDB ID: 3T07).

In each docking pose, catechin (**3**) scored higher binding energy than compound **2**. The docking results are presented in Tables 7–9. Comparatively, the binding score displayed by catechin (**3**) towards DNA gyraseB (−7.3) and *S. aureus* PK (−6.7) was found to be higher than that of the drug (−6.1 and −6.4), respectively (Tables 7–9). The binding score displayed by catechin (**3**) is in line with its largest *in vitro* antibacterial activity, which in turn support the previously reported bacterial activity of catechin [32]. Accordingly, Figures S4–S6 illustrate how residues interact to stabilize the ligand-protein interaction via H-bonding and other remaining interactions. H-bonding and the interactions are presented in the ball and stick model, and ligands are presented in various colors (Figures 5–7). Overall, the binding affinity of catechin (**3**) towards PqsA (−7.9 kcal/mol), despite being lower than that of amoxicillin (−8.3 kcal/mol), is however consistent with its *in vitro* test against *P. aeruginosa*.

### 3.5. Drug-Likeness and ADMET Properties

SwissADME tool was used to calculate physicochemical properties to compute the *in silico* drug resemblances and ADME parameters to assess molecular pharmacokinetics [28].

### 3.6. Drug-Likeness Properties

Based on the SwissADME prediction outcome (Table 10), compounds **2** and **3** obeyed all five rules of Lipinski (MW) < 500 Daltons, H-bond donors (NHD) < 5, H-bond, acceptors (NHA) < 10, and a log P of <5), implying their suitable drug resemblance properties. Compound **3** obeys Veber's rule with zero violation, indicating its structural stability. Both the studied compounds (**2** and **3**) scored LogP much less than five (3.35 and 0.85, respectively) implying their optimal lipophilicity. The predicted TPSA shows all three compounds (**1–3)** have TPSA less than 140 Å^2^, indicating better intestinal absorption properties. Compounds **1** and **2** scored TPSA values (37.3 and 86.99, respectively) much lower than the maximum value (140 Å^2^).

### 3.7. ADME Predictions

According to the ADME perdition result, the logKp value (human skin permeability coefficients) of isolated compounds (**1–3**) computed in the range −2.6 to −7.82 cm/s (Table 10), verifying its reduced skin permeability compared to amoxicillin (−9.94 cm/s). Compared to amoxicillin, compounds **1–3** displayed high GI absorption properties, inferring their favorable GI absorption property. No BBB permeation to CNS was predicted in each case. The lowest skin permeant was scored by compound **2** (−7.82 cm/s). Compounds **1** and **2** were predicted to be P-gp nonsubstrate (PGP-), having high GIA. Compound **3** was found to be P-gp substrate. No inhibitory effect was predicted by compound **1** towards CYP1A2 and CYP2C9, and compound **3** towards CYP2C9 indicating these isoforms may not be involved in the biotransformation of the corresponding noninhibiting compounds. Compound **2** was found to be noninhibitor of all selected cytochromes.

**Figure 8:**
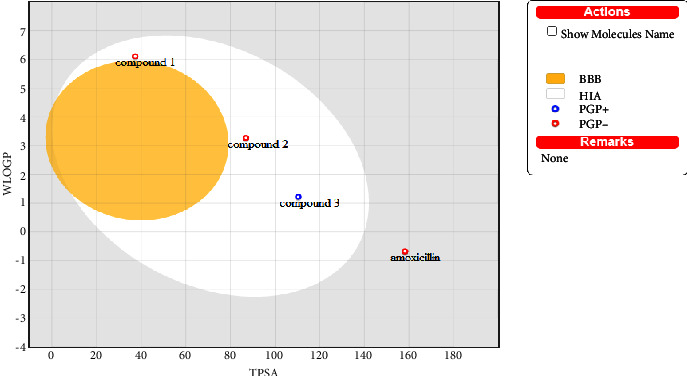
BOILED-Egg overview for brain access and intestinal absorption.

According to the BOILED-Egg prediction result (Figure 8), all isolated compounds (**1–3)** fall in the white region indicating the probability of being passively absorbed by the GIT, but none of the isolated compounds permeate the brain (out of the yellow region). Compounds **1** and **2** were detected as being non-PGP− (red spot) and hence not actively pumped up to the gastrointestinal lumen, whereas compound **3** was described as PGP+ (blue dot) and hence actively pumped up from the gastrointestinal lumen. Comparatively, the standard drug amoxicillin, which is located outside white or yellow ellipse, has low probability to permeate through the BBB to access the CNS and low absorption by the gastrointestinal tract.

### 3.8. Toxicity Prediction

Toxicity profiles of studied compounds (**1–3)** were computed by using the ProTox-II platform [33], and the results are displayed in Table 11. Caffeic acid-2-hydroxynonyl ester (**2**) and catechin (**3**) each scored LD_50_ greater than 5000 (class of toxicity less than five) indicating reduced acute toxicity of each. According to the obtained result, the examined compounds are inactive to induce hepatotoxicity, and in turn unlike to interrupt the normal duty of the liver. Compounds were estimated to be noncarcinogenic, mutagenic, and cytotoxic. Compound **2** exhibits an immunotoxicity effect. By taking their ADMET properties into consideration, compounds can be considered as a candidate to enter the early drug discovery stages with structural modifications.

## 4. Conclusion

In this study, four compounds, namely, oleic acid (**1**), caffeic acid-2-hydroxynonylester (**2**), catechin (**3**), and 3,9-epoxypregn-16-ene-14,20-diol,7,11,18-triacetoxy-3-methoxy (**4**), were identified from CH_2_Cl_2_/CH_3_OH extract of roots of the plant. Compounds **1**, **2**, and **4** were reported in this study for the first time from this plant. The *n*-hexane, CH_2_Cl_2_/CH_3_OH, and CH_3_OH filtrates showed cytotoxicity against MCF-7 lines. The *n*-hexane extract exhibited the strongest cytotoxicity at % cell viability of 28.9%. Catechin (**3**) exhibited strong activity with the highest zone of inhibition against *P. aeruginosa* followed by *E. coli*, good compared to amoxicillin. *In silico* molecular docking study findings also support the *in vitro* test. CH_2_Cl_2_/CH_3_OH extract displayed the largest radical trapping ability (70.32%) with IC_50_ value of 0.144 *μ*g/mL, and catechin (**3**) (65.61%), with IC_50_ value of 0.25 *μ*g/mL, relative to ascorbic acid (78.21%), with IC_50_ of 0.014 *μ*g/mL. Compounds **1**–**3** are predicted to acquire drug-like properties. All tested compounds (**1–3)** have large chance to be passively absorbed by the GIT, but none of the isolated compounds permeate. Catechin (**3**) actively refluxed (PGP+) and hence actively pumped up from the gastrointestinal lumen. Catechin (**3**) exhibited negligible acute toxicity effect. Thus, the observed cytotoxicity results of extracts and *in vitro* tests of the studied compounds corroborate with traditional uses of *H. johannis* in the treatment of infectious diseases and breast cancer. However, *in vivo* studies are needed to be carried out. The findings will also give insight into the potential use of the plant as a good source of lead compounds in drug development.

## Figures and Tables

**Table 1:** ^1^H NMR (400 MHz) and ^13^C NMR (100 MHz) spectra of compound **1**, CDCl_3_.

No	Compound 1		Reference [34]	
	^1^H	^13^C	^1^H	^13^C
1	—	179.6	—	180.5
2	2.31 (m, 2H)	34.0	2.51 (2H, t, H-2)	34.1
3	1.63 (m, 2H)	24.8	1.49 (2H, m, H-3)	24.8
4–7	2.01 (m, 2H)	29.0–29.4	2.04 (2H, m, H-4)	29.0–29.6
8, 11	1.83 (m, 2H)	27.2	1.97 (2H, m, H-8)	27.2
9, 10	5.33 (m, 1H)	129.8, 130.1	5.33 (1H, d)	129.7, 130.0
12–15	2.01 (m, 2H)	29.3–29.5	2, 04 (2H, m, H-12)	29.3–29.5
16, 17	1.83 (m, 2H)	31.9, 22.7	2.19 (2H, m, H-16)	31.9, 22.6
18	0.99 (m, 3H)	14.2	0.96 (3H, t, H-18)	14.3

**Table 2:** ^1^H (400 MHz) and ^13^C NMR (100 MHz) spectra of compound **2**, CDCl_3_.

No	Compound 2		Reference [35]	
	^1^H	^13^C	^1^H	^13^C
1		128.9		127.9
2	7.05 (d, *J* = 2.0 Hz, 1H)	114.7	7.05, br, s	115.3
3		146.7		146.9
4		147.9		149.5
5	6.92 (d, *J* = 8.1 Hz, 1H)	115.6	6.78, d, 8.0	115.0
6	7.08 (d, *J* = 8.1 Hz, 1H)	123.05	6.95, br, d, 8.0	123.1
7	7.62 (d, *J* = 15.9 Hz, 1H)	144.7	7.58, d, 16.0	147.2
8	6.30 (d, *J* = 15.9 Hz, 1H)	115.8	6.29, d, 16.0	116.3
9		167.5		169.4
1′	4.34/4.28 (dd, *J* = 12.0, 4.4 Hz, 2H)	65.14	4.26/4.16 (dd, 4.0, 11.2)	66.7
2′	4.15 (m, 1H)	68.99		71.4
3′	2.31 (m, 2H)	34.1		
4′		29.1		
5′		27.1		
6′		24.8		
7′		31.9		
8′		22.7		
9′	0.89 (t, *J* = 6.8 Hz, 3H)	14.1		

**Table 3:** ^1^H (400 MHz) and ^13^C NMR (100 MHz) spectra of compound **3**, CD_3_OD.

No	Compound 3		Reference [36]
	^1^H	^13^C	^13^C
2	4.46 (d, *J* = 7.9 Hz, 1H)	81.3	81.4
3	3.88 (m, 1H)	67.4	66.8
4	2.81 (dd, *J* = 16.0, 5.5 Hz, 1H), 2.41 (dd, *J* = 16.0, 5.5 Hz, 1H)	27.3	28.2
5		155.7	156.6
6	5.81 (d, *J* = 1.2 Hz, 1H)	95.3	95.6
7		155.9	156.9
8	5.84 (d, *J* = 1.2 Hz, 1H)	94.6	94.4
8a		155.3	155.8
4a		99.3	99.6
1′		130.2	131.1
2′	6.74 (d, *J* = 1.9 Hz, 1H)	114.1	114.9
3′		144.6	145.3
4′		144.4	145.3
5′	6.68 (d, *J* = 8.1 Hz, 1H)	115.1	115.6
6′	6.65 (dd, *J* = 8.2, 1.9 Hz, 1H)	119.2	118.9

**Table 4:** Cytotoxicity of *C. cyphopetalum*, *H. johannis*, and *C. tomentosa* extracts against MCF-7 cell line at 20 *µ*g/mL.

Samples	% of cell viability	SD	Estimated IC_50_ (*μ*g/mL)
Control	100.0	0.14	
*n*-Hexane extract	28.9	0.19	<20
DCM/MeOH extract	59.5	0.15	
MeOH extract	51.6	0.28	20

**Table 5:** Zone of inhibition bacterial growth by compounds **1**, **2**, and **3**.

Samples	Concentration	Strains			
		*E. coli*	*P. aeruginosa*	*S. aureus*	*S. pyogen*
**1**	0.25	7.35 ± 0.15	7.89 ± 0.05	7.10 ± 0.49	8.13 ± 0.19
	0.5	9.71 ± 0.06	10.41 ± 0.48	8.95 ± 0.05	9.62 ± 0.12
**2**	0.25	9.19 ± 0.06	10.32 ± 0.12	8.97 ± 0.22	8.2 ± 0.3
	0.5	11.04 ± 0.05	11.87 ± 0.97	10.00 ± 0.89	10.15 ± 0.05
**3**	0.25	12.25 ± 0.1	13.72 ± 0.05	10.20 ± 0.21	11.62 ± 0.12
	0.5	13.89 ± 0.09	14.8 ± 0.2	10.82 ± 0.05	12.66 ± 0.05
Amoxa (0.25 mg/mL)		15.25 ± 0.25	16.0 ± 0.0	15.25 ± 0.25	16.25 ± 0.25

Samples were reported as mean ± SD (in mm); replicates (N) = 2; concentrations in (mg/mL).

**Table 6:** Percentage inhibition (mean ± SD) of the DPPH radical by isolated compounds **1**–**3**.

Samples	% DPPH inhibition (*μ*g/mL)					IC_50_
Extracts	200	100	50	25	12.5	
n-hexane	62.06 ± 0.03	58.69 ± 0.01	53.37 ± 0.04	49.33 ± 0.01	44.86 ± 0.03	1.85
DCM/MeOH	87.09 ± 0.02	83.41 ± 0.02	78.09 ± 0.01	75.89 ± 0.01	70.32 ± 0.01	0.144
MeOH	85.43 ± 0.01	75.40 ± 0.01	73.32 ± 0.01	70.01 ± 0.01	67.44 ± 0.03	0.056

*Compounds*						
**1**	55.32 ± 0.04	51.35 ± 0.05	50.31 ± 0.04	45.04 ± 0.05	43.64 ± 0.04	2.94
**2**	66.86 ± 0.01	60.77 ± 0.04	56.67 ± 0.09	52.63 ± 0.004	48.90 ± 0.01	1.31
**3**	82.99 ± 0.01	76.01 ± 0.01	72.09 ± 0.01	68.18 ± 0.02	65.61 ± 0.004	0.25
Ascorbic acid	90.52 ± 0.03	82.17 ± 0.03	80.16 ± 0.02	79.53 ± 0.02	78.21 ± 0.03	0.0004

**Table 7:** Binding score of caffeic acid-2-hydroxynonyl ester (**2**) and catechin (**3**) against DNA gyraseB.

Compounds	Binding energy	H-bonding	Interactions	
			Hydrophobic/electrostatic	Van der Waals
**2**	−5.4	Gly-77, Asn-46, and Asp-73	Glu-50, Ala-53, and Ile-78	Ileu-94, Pro-79, Gly-75, Gly-164, Thr-165, Arg-76, and Asp-49
**3**	−7.3	Asp-73, Gly-77, and Arg-76	Glu 50, Pro-79, and Ile-78	Thr-165, Gly-75, Ala-47, Asn-46, and Ile-94
Amoxicillin	−6.1	Val-120, Ser-121, Ile-94, Val-97, Glu-50, and Gly-119		

In all cases, binding energy is in kcal/mol.

**Table 8:** Binding score of caffeic acid-2-hydroxynonyl ester (**2**) and catechin (**3**) against PqsA.

Compounds	Binding energy	H-bonding	Interactions	
			Hydrophobic/electrostatic	Van der Waals
**2**	−5.3	Ala-21, Arg-200, Thr-29, Ala-245, and Arg-247	Arg-200, Arg-249, and Arg-247	Asp-199, Asp-17, Thr-20, Leu-30, Gln-34, Val-244, Ser-31, Asp-19, Ser-226, His-24, and Gly-198
**3**	−7.9	Arg-333, Arg-386, Tyr-392, Glu-343, and Cys-332	Arg-186 and Arg-333	Pro-326, Leu-325, Gly-390, Glu-191, Pro-328, Ala-187, Gly-329, Pro-356, Glu-331, and Thr-341
Amoxicillin	−8.3	Arg-186, Arg-333, Arg-386, Glu-343, and Glu-331	Arg-333	Arg-179, Thr-341, Ile-342, Leu-182, Gly-183, Tyr-392, Cys-332, and Trp-377

**Table 9:** Binding score of caffeic acid-2-hydroxynonyl ester (**2**) and catechin (**3**) against *S. aureus* PK.

Compounds	Binding energy	H-bonding	Interactions	
			Hydrophobic/electrostatic	Van der Waals
**2**	−5.4	Asp-339, Tyr-302, and Ala-337	Arg-264, Asp-346, and Tyr-302	Gly-338, Leu-343, Gly-304, Asp-303, Lys-260, Lys-342, Asn-299 B, and Asn-299 C
**3**	−6.7	Leu-269, Asp-237, Gly-483, and Glu-234	Arg-386 and Lys-271	Ser-236, Ile-482, Arg-484, Lys-390, Gly-270, and Pro-272
Amoxicillin	−6.4	Arg-204, Lys-271, and Arg-386	Arg-386 and Ser-383	Val-235, Glu-234, Gly-483, Ala-573, Thr-387, Val-356, Thr-464, Thr-463, Leu-269, Asp-237, and Ser-236

**Table 10:** Drug likeness and ADME values of compounds **1**, **2**, and **3**.

Computed properties	Compounds			Amoxicillin
Drug likeness	1	2	3	
Formula	C_18_H_34_O_2_	C_18_H_26_O_5_	C_15_H_14_O_6_	C_16_H_19_N_3_O_5_S
MW (g/mol)	282.46	322.4	290.27	365.40
NHD	1	3	5	4
NHA	2	5	6	6
LogP	5.65	3.35	0.85	−0.3
Lipinski's RO5 violation	1	0	0	0

NRB	15	11	1	5
TPSA (Å^2^)	37.3	86.99	110.38	158.26
Veber's rule violation	1	1	0	1

*ADME properties*				
logKp (cm/s)	−2.6	−7.82	−4.72	−9.94
GI absorption	High	High	High	Low
BBB permeation	No	No	No	No

*Inhibitions*				
P-gp substrate	No	Yes	No	No
CYP1A2 inhibitor	Yes	No	No	No
CYP2C19 inhibitor	No	No	No	No
CYP2C9 inhibitor	Yes	No	Yes	No
CYP2D6 inhibitor	No	No	No	No
CYP3A4 inhibitor	No	No	No	No

**Table 11:** Toxicity profile of compounds **1**, **2**, and **3**.

Compounds	LD_50_ in (mg/kg)	Class of toxicity	Toxicity				
			Hepat	Carcin	Immun	Muta	Cyto
1	48	2	Non	Non	Non	Non	Non
2	9600	6	Non	Non	Active	Non	Non
3	10000	6	Non	Non	Non	Non	Non

Non = inactive.

## Data Availability

All data generated or analyzed during this study are included within the article and supplementary materials.

## Supplementary Materials

Supplementary Materials The NMR spectra used to establish the structures of the compounds isolated in this work are depicted as Figures S1-S9. Figure S1: ^1^H NMR spectrum of compound 1 (400 MHz, CDCl_3_). Figure S2. ^13^C NMR spectrum of compound 1 (100 MHz, CDCl_3_). Figure S3: DEPT-135 NMR spectrum of compound 1 (100 MHz, CDCl_3_). Figure S4: ^1^H NMR spectrum of compound 2 (400 MHz, CDCl_3_). Figure S5: ^13^C NMR spectrum of compound 2 (400 MHz, CDCl_3_). Figure S6: DEPT-135 NMR spectrum of compound 2 (400 MHz, CDCl_3_). Figure S7: ^1^H NMR spectrum of compound 3 (400 MHz, CDCl_3_). Figure S8: ^13^C NMR spectrum of compound 3 (100 MHz, CDCl_3_). Figure S9: DEPT-135 NMR spectrum of compound 3 (400 MHz, CDCl_3_).
