# Supplementary material for: In Vitro Antibacterial, Antioxidant, Cytotoxicity Activity, and In Silico Molecular Modelling of Compounds Isolated from Roots of Hydnora johannis
Source: Biochem Res Int. 2024 Jun 21;2024:3713620. doi: 10.1155/2024/3713620 (PMC11213641; doi:10.1155/2024/3713620)
Supplement: Supplementary Materials — The NMR spectra used to establish the structures of the compounds isolated in this work are depicted as Figures S1-S9. Figure S1: 1H NMR spectrum of compound 1 (400 MHz, CDCl3). Figure S2. 13C NMR spectrum of compound 1 (100 MHz, CDCl3). Figure S3: DEPT-135 NMR spectrum of compound 1 (100 MHz, CDCl3). Figure S4: 1H NMR spectrum of compound 2 (400 MHz, CDCl3). Figure S5: 13C NMR spectrum of compound 2 (400 MHz, CDCl3). Figure S6: DEPT-135 NMR spectrum of compound 2 (400 MHz, CDCl3). Figure S7: 1H NMR spectrum of compound 3 (400 MHz, CDCl3). Figure S8: 13C NMR spectrum of compound 3 (100 MHz, CDCl3). Figure S9: DEPT-135 NMR spectrum of compound 3 (400 MHz, CDCl3). [file 3713620.f1.docx]

**Supporting information**

***In vitro* Antibacterial, Antioxidant, Cytotoxicity Activity and *In silico* Molecular modelling of Compounds Isolated from roots of *Hydnora johannis***

**Teshome Degfie^1,5^, Milkyas Endale^1^*, Muhdin Aliye^1^, Rajalakshmanan Eswaramoorthy^2^, Tariku Nefo^3^, Aman Dekebo^1,4^**

**Corresponding authors:** [amandekeb@gmail.com](mailto:amandekeb@gmail.com), [milkyas.endale@astu.edu.et](mailto:milkyas.endale@astu.edu.et)

The NMR spectra used to establish the structures of the compounds isolated in this work are depicted as Fig. S1-S9.

Figure S1: ^1^H NMR spectrum of compound **1 (**400 MHz, CDCl_3_).

Figure S2. ^13^C NMR spectrum of compound **1 (**100 MHz, CDCl_3_).

Figure S3: DEPT-135 NMR spectrum of compound **1 (**100 MHz, CDCl_3_).

Figure S4: ^1^H NMR spectrum of compound **2 (**400 MHz, CDCl_3_).


Figure S5: ^13^C NMR spectrum of compound **2 (**400 MHz, CDCl_3_)


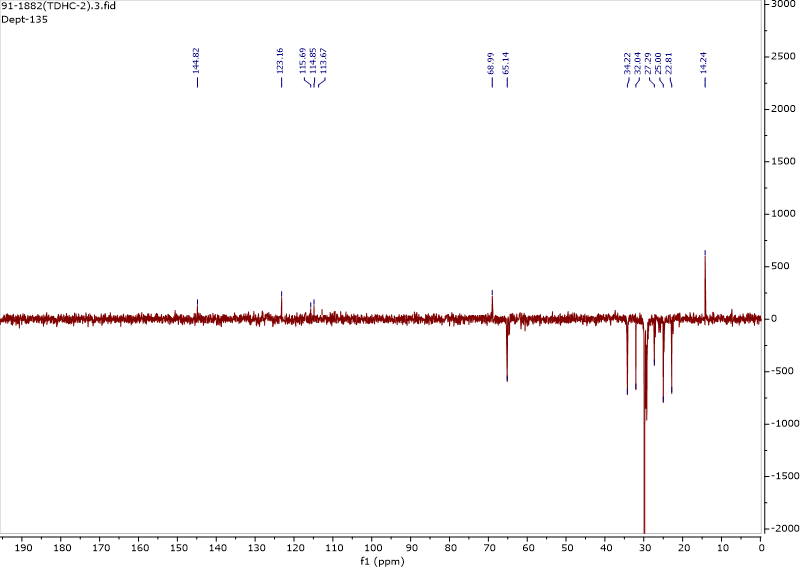
Figure S6: DEPT-135 NMR spectrum of compound **2 (**400 MHz, CDCl_3_).

Figure S7: ^1^H NMR spectrum of compound **3 (**400 MHz, CDCl_3_).

Figure S8:^13^C NMR spectrum of compound **3 (**100 MHz, CDCl_3_).

Figure S9: DEPT-135 NMR spectrum of compound **3 (**400 MHz, CDCl_3_).
